# Spectral characteristics of voltage-sensitive indocyanine green fluorescence in the heart

**DOI:** 10.1038/s41598-017-08168-7

**Published:** 2017-08-11

**Authors:** Regina Mačianskienė, Mantė Almanaitytė, Rimantas Treinys, Antanas Navalinskas, Rimantas Benetis, Jonas Jurevičius

**Affiliations:** Institute of Cardiology, Lithuanian University of Health Sciences, Kaunas, Lithuania

## Abstract

Indocyanine green (ICG) fluorescent dye has been approved by the FDA for use in medical diagnostics. Recently, we demonstrated that ICG dye has voltage-sensitive properties with a dual-component (fast and slow) response in the Langendorff-perfused rabbit heart. Here, we extended our studies by showing the different spectral properties of both components for analysis of the fractional change in ICG fluorescence in response to voltage changes. We used light from four LEDs to obtain excitation; emission was measured using an EMCCD camera with band-pass filters and a spectrometer. We applied a graphical model with Gaussian functions to construct and evaluate the individual emission curves and calculated the voltage-sensitive portion of each component of the ICG fluorescence in the rabbit heart. The results revealed that each isolated component (fast and slow) emanates from a unique ICG pool in a different environment within the cell membrane and that each component is also composed of two constituents (ICG-monomeric and ICG-aggregated). We propose the existence of different voltage-sensitive mechanisms for the components: (I) electrochromism and field-induced reorientation for the fast component; and (II) field-induced dye squeezing that amplifies intermolecular interactions, resulting in self-quenching of the dye fluorescence, for the slow component.

## Introduction

To date, voltage-sensitive fluorescent dyes (VSDs) have been used only in experimental studies because most VSDs have not yet been approved for clinical use. Indocyanine green (ICG) dye is the only voltage-sensitive fluorescent dye^1^ that has obtained FDA approval for use in medical diagnostics. Our group reported the first successful detection of voltage sensitivity of a near-infrared ICG dye in a rabbit heart^2^. We also showed that the optical signal (OS) obtained with this dye has a dual-component (fast and slow) response to membrane potential changes that accurately tracks the time of electrical signal propagation but that the two components clearly differ in their kinetics and may be either positive or negative in different parts of the fluorescence spectrum under the same experimental conditions. Overall, the voltage-sensitive fluorescence of ICG dye in a whole-heart preparation that was fully stopped was not high relative to the fluorescence of standard voltage-sensitive styryl dyes^3^. However, after averaging, the good signal-to-noise ratio (>20 dB) of ICG rendered its signal suitable for observing cardiac electrical activity. In addition, we showed that the OS obtained using ICG is not caused by contraction or by Ca^2+^ transients, that it reliably follows the electrical action potential (AP) changes induced by pharmacological compounds, and that it is suitable for use in evaluating AP activation time and cardiac electrical conduction. Accordingly, we proposed that separating the OS into two independent components revealed two different mechanisms for the voltage sensitivity of the ICG dye. It was tempting to speculate that the fast component (OS_f_) is most likely electrochromic in nature^4^, whereas the slow component (OS_s_) might be explained by a repartitioning mechanism^5, 6^. However, the actual situation is unclear because the ICG molecule, unlike classical electrochromic dyes, is symmetrical and contains two negative charges located at opposite ends of the molecule. Therefore, on the one hand, the ICG molecule should have zero dipole moment along its long axis and should not exhibit electrochromism^7^. On the other hand, the ICG dye has been shown to have solvatochromic properties^8, 9^; thus, the dipole moment of the dye apparently is not zero, suggesting that it should also exhibit electrochromism.

Against this background, we aimed both to conduct a more detailed investigation of the voltage-sensitive fluorescence of the ICG OS components and to determine their fluorescence spectra. The emission properties of ICG fluorescence in the rabbit heart have been investigated, and the fluorescence of the dye in liquid solutions supplemented with proteins or lipids has also been studied. This study focused on relatively low ICG concentrations (in the range of 0.03–10 µM) because higher dye concentrations elicit disturbances in AP formation and propagation in the heart. In agreement with the results of our previous study^2^, we confirmed both that the ICG fluorescent dye has voltage-sensitive properties in a Langendorff-perfused rabbit heart and that the voltage-sensitive reaction of ICG includes two components with different time constants. In addition, we revealed that each isolated component (fast and slow) has different voltage-sensitive spectral properties and is composed of two constituents, possibly because of the existence of ICG monomers and ICG aggregates.

We propose that distinct spectral properties might arise from different pools of ICG within the cell membrane when the fluorescent dye is located in environments that contain membrane proteins or phospholipids. Therefore, separate pools could form voltage-sensitive components with different kinetics, spectral properties, and voltage-sensitive mechanisms.

## Materials and Methods

### Experimental preparation

All of the experimental procedures involving animals conformed to the European Community’s guiding principles and were approved by the State Food and Veterinary Service of the Republic of Lithuania (2015-09-24 No.G2-34).

Experiments were performed on Langendorff-perfused hearts from New Zealand White rabbits of both sexes weighing 3.8 ± 0.1 kg (n = 9). The details of the anesthesia and heart-excision procedures have been described previously^2, 3^. The heart was quickly cannulated through the aorta and attached to a Langendorff perfusion system with a constant pressure of ~80 mmHg and perfused with oxygenated (100% O_2_) Tyrode’s solution (in mM: 135 NaCl, 5.4 KCl, 1.8 CaCl_2_, 0.9 MgCl_2_, 0.33 NaH_2_PO_4_, 10 glucose, and 10 HEPES; pH 7.4) at 37 ± 0.5 °C. The coronary flow rate was 39.1 ± 0.6 mL/min. After allowing approximately 20–30 minutes for the preparation to equilibrate, the perfusion was switched to recirculation mode. Blebbistatin (20 µM) and 2,3-butanedione monoxime (BDM, 5 mM) were added to the perfusate to suppress contractions while allowing electrical activity to continue. The heart was stained by adding increasing concentrations (from 0.03 µM to 10 µM) of the ICG dye to the perfusate.

The hearts were paced at a 300-ms period from the endocardial surface of the LV (close to apex) via a bipolar silver electrode with a 2-ms impulse at twice the diastolic threshold.

### Optical measurements

The optical mapping (OM) technique for measuring the voltage sensitivity of ICG fluorescence in Langendorff-perfused hearts has been described previously^2^. In brief, we used collimated LEDs (Thorlabs, USA) for excitation (617 nm, 660 nm, 735 nm, and 780 nm; filtered at the corresponding wavelengths with band-pass filters; see Supplementary Table S1). Emission was measured using band-pass and long-pass filters for various wavelengths (from 700 nm to 905 nm; see Supplementary Table S2) with a cooled (−100 °C) fast 14-bit EMCCD camera (iXon^EM+^DU-860, Andor Technology, Ireland) equipped with a 50-mm focal length objective (Navitar, USA) and with a spectrometer (from 350 nm to 1020 nm, Ocean Optics, flame-S-VIS-NIR, USA). The anterior surface of the LV was imaged, and the field of view was 20 × 20 mm. Optical movies were acquired at a sampling rate of 500 Hz with a resolution of 128 × 128 pixels using imaging software (Andor SOLIS x-3467). To mark the time of electrical stimulation in the optical recording, an array of small LEDs (700–940 nm) that generated 2-ms pulses in synchrony with the pacing electrical pulse was placed in the camera’s field of view. All of the experiments were performed under dark conditions, avoiding the exposure of the preparation to light.

### ICG fluorescence detection

Optical signals recorded with a spectrometer were collected for periods of 3 sec in duration. The dye-free signal was subtracted from the fluorescence signal recorded at all tested dye concentrations. Under such conditions, no fluorescence difference for the resting potential (RP) and the AP was detected. Thus, taking into account that the voltage-sensitive portion of the ICG OS is only ~1%, the entire fluorescence could be taken as basal fluorescence and will correspond to the RP (hereinafter referred to as F). The AP (hereinafter referred to as ΔF) - induced fluorescence changes that occur under such circumstances are not evaluated.

In cases in which the fluorescence signals were recorded with a fast EMCCD camera, the F and the ΔF were isolated. Each recording was performed for 10 seconds. To decrease the noise, 32 OSs were averaged, and for the analysis, signals were taken from an area of 15 × 40 pixels. The optical movies were pre-processed using ImageJ software. The dye-free fluorescence level obtained at all LEDs and with all emission filters was subtracted from every OS stack of the recording made in the presence of the dye. Because of the time required to save the data and change the LEDs and emission filters, the time period required for each individual recording was 20–30 seconds.

The ICG fluorescence data obtained using a standard EMCCD camera and a broad set of emission filters include all of the necessary information about not only the time and spectrum features of the OS but also the properties that characterize the dye’s voltage sensitivity. However, because the various emission filters used in this study had different characteristics (e.g., light transmission and spectral width), we encountered difficulties in constructing a reliable fluorescence spectrum.

It should certainly be noted that these spectra could easily be recorded with a standard spectrometer. Therefore, the use of both an EMCCD camera with an appropriate set of emission filters and a spectrometer in the same experiment made it possible to determine the fluorescence spectrum of the ICG dye and evaluate the sensitivity of the dye’s spectral properties to voltage in a simple and straightforward manner.

The procedure for detection of background fluorescence (F) and construction of fluorescence spectra depending on voltage was as follows. First, the ICG fluorescence obtained experimentally using a fast EMCCD camera was used to calculate the time course of a voltage-sensitive portion of that signal by subtracting the background fluorescence value from the whole optical signal (i.e., the OS at rest was set to zero). Second, by applying ratiometric and multiplication procedures^2^ the signal was divided into two components (fast and slow), and their maximal values were calculated. In this manner, three different values were obtained from the ICG fluorescence signal recorded with a fast EMCCD camera: F, ΔF_f_ and ΔF_s_. These values were obtained using each emission filter at the corresponding wavelength. In the next step, the ICG fluorescence signals recorded with the spectrometer were normalized to 100% at each LED and dye concentration. From the normalized fluorescence spectrum, a section of a predefined width that corresponded to each of the fluorescence filters used with the EMCCD camera was extracted. This enabled calculation of the mean value of the fluorescence intensity in the extracted sections. The averaged values were assigned as the background fluorescence (F) obtained with an EMCCD camera. The corresponding values for ΔF_f_ and ΔF_s_ were adjusted proportionally.

### Fluorescence detection in the presence of albumins and lipids

Fluorescence spectra were recorded with a spectrometer using a right-angle configuration^10^. Briefly, in this study we used Tyrode’s solution containing either albumin (1 mg/mL) or lipids (0.75 mg/mL). The prepared suspension was poured into a cuvette, placed on a magnetic heating plate, and stirred continuously in the dark at a temperature of 37 °C. Experiments were performed when the cuvette was subsequently illuminated with LEDs (see above).

### Analysis of OS

Overall, our analysis was directed at the characterization of two components, OS_f_ and OS_s_, of the ICG VSD described in our earlier study^2^. We hypothesized that each component has a separate and distinct fluorescence spectrum.

The skew Gaussian functions were the most frequently used in analysis of the voltage sensitivity of the fluorescence of VSDs^11, 12^. Our initial analysis (not shown) indicates that the two skew Gaussian spectra, one each for the fast and slow components, are not ideal for evaluating changes in the emitted fluorescence of the ICG dye over the entire fluorescence spectrum range, particularly for voltage-sensitive OS at red tail. Also, it is not possible to evaluate the reason for skewing (i.e., asymmetry) of the results. Therefore, to describe the complex response of the ICG dye to changes in the membrane potential, four symmetrical Gaussian functions of a three-parameter Gaussian lineshape function were used (two for the OS_f_ and two for the OS_s_ component). This choice proved well suited for describing the experimental data over the entire range of the ICG dye fluorescence spectrum (see Results). On the one hand, the four symmetrical Gaussian functions permitted a good approximation of the experimental data points both for the total emitted fluorescence and for its voltage-sensitive portion. On the other hand, the model was consistent with the possibility that two of those functions were formed by the aggregation of the dye. Apparently, at each of the components one spectrum could be related to the ICG-monomer form, whereas the other is associated with the ICG-aggregate form. Although this does not support our earlier suggestion that there is no aggregation^2^ at the ICG dye concentration used in the study, only after a detailed assessment of the emission spectrum was it possible to determine the existence of aggregation at the ICG dye concentrations used in this study.

We assumed that each component has two constituents: two for OS_f_ (f1, f2) and two for OS_s_ (s1, s2); both components were approximated using the four symmetrical three-parameter Gaussian functions. The Gaussian lineshape function can be expressed as follows:

1 $${\rm{A}}\,\exp \{-[{({{\rm{\lambda }}-{\rm{\lambda }}}_{{\rm{\max }}})}^{2}/(2\,{\mathrm{HW}}^{2})]\},$$

where A is the fluorescence amplitude, λ is a wavelength, λ_max_ is the wavelength at the fluorescence maximum, and HW is the half width of the spectrum (detected at 50% of the fluorescence maximum).

To construct four fluorescence spectral lineshapes, three different values were measured for each constituent (f1, f2, s1, s2): the background fluorescence at the resting potential (F_f1_, F_f2_, F_s1_, F_s2_, respectively); the fluorescence at depolarization, i.e., at the maximal value of AP (F_f1_ + ΔF_f1_, F_f2_ + ΔF_f2_, F_s1_ + ΔF_s1_, F_s2_ + ΔF_s2_, respectively); and the fractional fluorescence, i.e., the voltage-sensitive portion (ΔF_f1_, ΔF_f2_, ΔF_s1_, ΔF_s2_, respectively).

### Chemicals

(±)-Blebbistatin, 2,3-butanedione monoxime (BDM), ICG (Cardiogreen), bovine serum albumin (BSA), and a mixture of phospholipids (Asolectin Soybean) were obtained from Sigma-Aldrich (USA). A stock solution of the ICG dye (1 mg/mL in distilled water) was freshly prepared prior to each experiment and diluted to various concentrations (0.03–10 µM) in Tyrode’s solution just before use.

### Statistics

Data are presented as the mean ± standard error of mean (s.e.m.). The significance of differences was evaluated using one-way analysis of variance (ANOVA). The significance level was set at *p* < 0.05.

## Results

### Spectrum of ICG fluorescence in the heart

Our recent study^2^ revealed both that ICG displays voltage-sensitive properties in the heart and that the ICG OS includes fast and slow components that differ in their kinetics. However, the fluorescence spectral properties of the voltage sensitivity of the ICG dye in the heart were not investigated in that study. Therefore, it remained unclear whether the emission spectra of the fast and slow components also differ. This prompted us to investigate the spectral properties of the voltage sensitivity of ICG in detail. For this, the background spectral properties and the fractional fluorescence (i.e., the voltage-sensitive portion) of both components must be analyzed. To accomplish this in our experiments, we recorded the OSs with a spectrometer to obtain a spectrum of the background fluorescence and in parallel used a fast EMCCD camera that records a fluorescence signal that is the sum of the background and the fractional fluorescence. This joint analysis allowed us to measure both the spectrum of the background fluorescence and the spectrum of the voltage-sensitive portion of each component of the OS.

**Figure 1:**
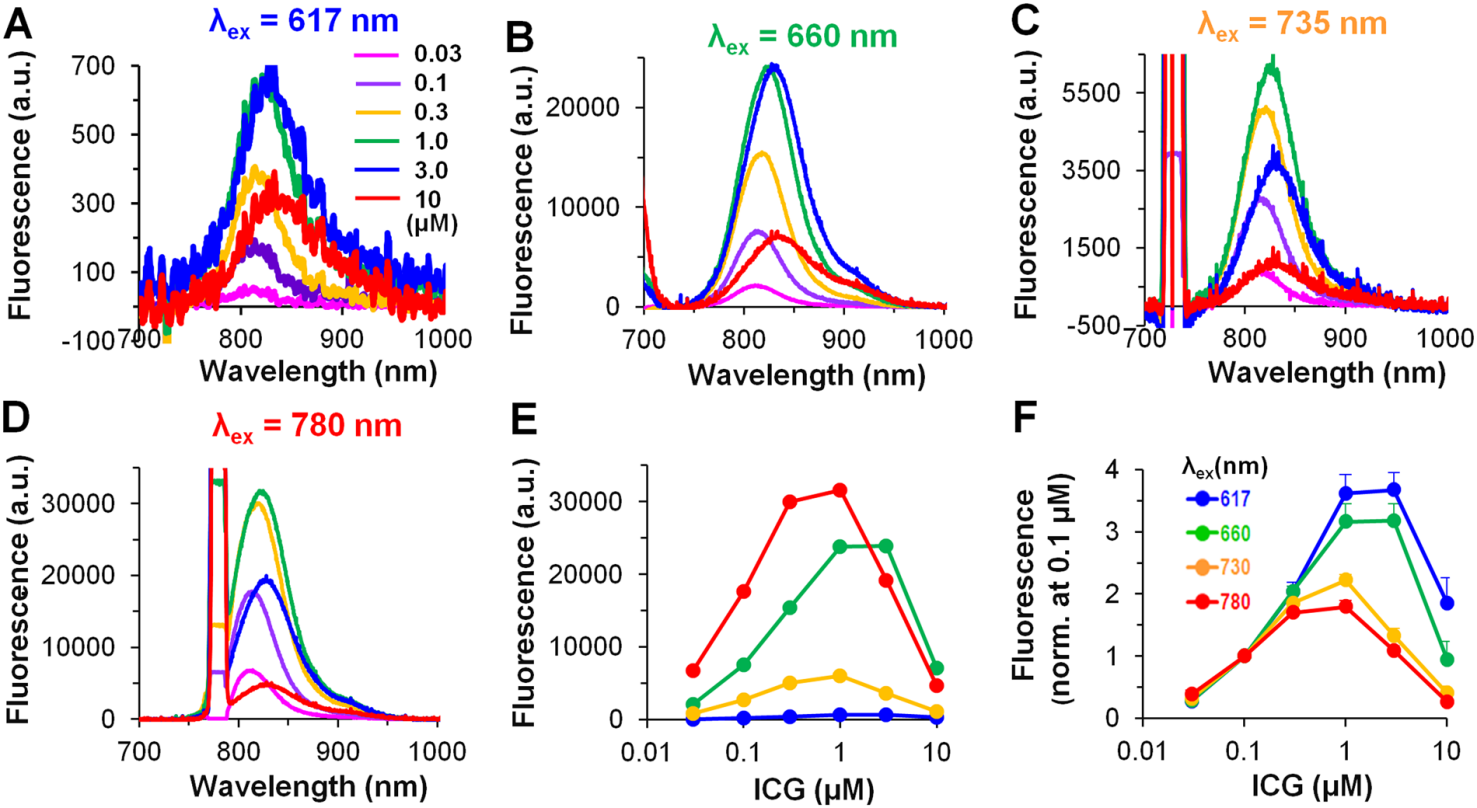
Fluorescence spectra of the ICG OSs as a function of dye concentration in the heart. (**A**–**D**) Mean values (n = 3–7) of ICG fluorescence at λ_ex_ of 617 nm, 660 nm, 735 nm, and 780 nm, respectively. (**A**) Insert - ICG dye concentrations as indicated in µM; (**B**–**D**) same labels as in (**A**). (**C,D**) on the left side of the spectra, the LEDs excitation light is seen. (**E,F**) Emission peaks at increased concentrations of the ICG dye: fluorescence intensity in arbitrary units (a.u.), and after fluorescence normalization (at 0.1 µM of the ICG), respectively; same fluorescence data as in (**A–D**). (**F**) Insert - λ_ex_ = 617 nm (*blue*), λ_ex_ = 660 nm (*green*), λ_ex_ = 735 nm (*yellow*), and λ_ex_ = 780 nm (*red*); (**E**) same labels as in (**F**). Note the difference in the fluorescence intensity when ICG concentrations > 0.3 µM were used. Error bars indicate s.e.m.

Initially, the effect of increasing the concentration of the ICG dye on its fluorescence spectrum was investigated using a spectrometer. The fluorescence of ICG OSs in the heart was studied as a function of light exposure at LED excitation peaks (λ_ex_) of 617 nm, 660 nm, 735 nm and 780 nm and at ICG dye concentrations ranging from 0.03 to 10 µM over a typical emission spectrum (from 700 nm to 1000 nm) for ICG fluorescence (Fig. 1A–D). Figure 1E,F, shows the dose-dependence of the mean peak values of fluorescence intensity (n = 3–7). Note the increase in the intensity at low concentrations of the dye and the decrease at high concentrations.

According to the literature, ICG dye can exist either in a monomeric form or in an aggregated form, and the quantum yield of the aggregated form of the dye is markedly reduced^13^. Therefore, the reduction in the emission spectrum at the high ICG dye concentrations may be caused by intermolecular interaction and aggregation of the dye molecules. Furthermore, the inner-filter effect could be additionally responsible for the decrease in the ICG fluorescence intensity at higher dye concentrations^14^.

Figure 1E,F, presents the maximal values of the emission spectrum. This permits graphical representation of both the ICG-monomeric and the ICG-aggregated fluorescence regions at different concentrations of the dye (0.03–10 µM) in the heart. However, without normalization, comparison of such values was problematic, particularly because of the different LED parameters. Therefore, the fluorescence data were normalized to fluorescence value at 0.1 µM dye concentration (Fig. 1F). It is known^15^ that at the higher excitation wavelengths (~780 nm) only the ICG-monomers could be excited. While at the lower excitation wavelengths (<700 nm) both the ICG-monomers and their aggregates could be excited. Hence, as shown in Fig. 1F, even at the low concentration of 1 µM there is a divergence in the data at λ_ex_ = 617 nm (3.62 ± 0.29) and λ_ex_ = 660 nm (3.16 ± 0.28) compared to the data obtained at λ_ex_ = 735 nm (2.22 ± 0.09) and λ_ex_ = 780 nm (1.79 ± 0.10) (n = 7 for each), possibly because some of the ICG monomers aggregated^16^ under these conditions. Thus, at higher concentrations, the ICG may be present not only in the monomeric form but also in the aggregated form (note the difference in the fluorescence intensity at low and high dye concentrations). We also suggest that the inner-filter effect caused by reabsorption of the emitted light has a greater influence in the case of the ICG-monomeric form than in the case of the ICG-aggregate form because the excitation and emission spectral peaks for the monomer are close to one another; at higher concentrations, this further reduces the fluorescence at excitation wavelengths of 735 nm and 780 nm. Additionally, as can be seen in Fig. 1A–D, the maximum peak of the spectrum (λ_max_) was shifted to the right when the dye concentration was increased. In most cases, the shift of the λ_max_ values is easier to compare when the data are normalized. Because of the presence of a big noise, the normalized data are not presented here; however, the normalized spectra approximated with Gaussian functions are shown in Supplementary Fig. S1.

### Splitting of ICG fluorescence into its constituents

From Fig. 1, it can be seen that the fluorescence spectrum has a characteristic bell-shaped curve. In other studies, the spectral analysis of VSDs was most commonly performed by applying a skew Gaussian lineshape profile^11, 12^. On the one hand, skewness in the Gaussian lineshape can be produced by adding a parameter of asymmetry as a fourth parameter to the three standard Gaussian parameters (i.e., the amplitude, the location of the peak and the width of the peak at 50% of the amplitude). On the other hand, the asymmetry can be produced as a sum of several Gaussian functions with different parameters. Figure 1 shows that the obtained fluorescence spectrum is also asymmetrical. Considering that in our study the obtained spectra could be formed as the sum of the fluorescence spectra of ICG-monomeric and ICG-aggregated forms whose spectral properties are distinct, we used the sum of symmetrical Gaussian functions to describe the asymmetry (see Methods).

**Figure 2:**
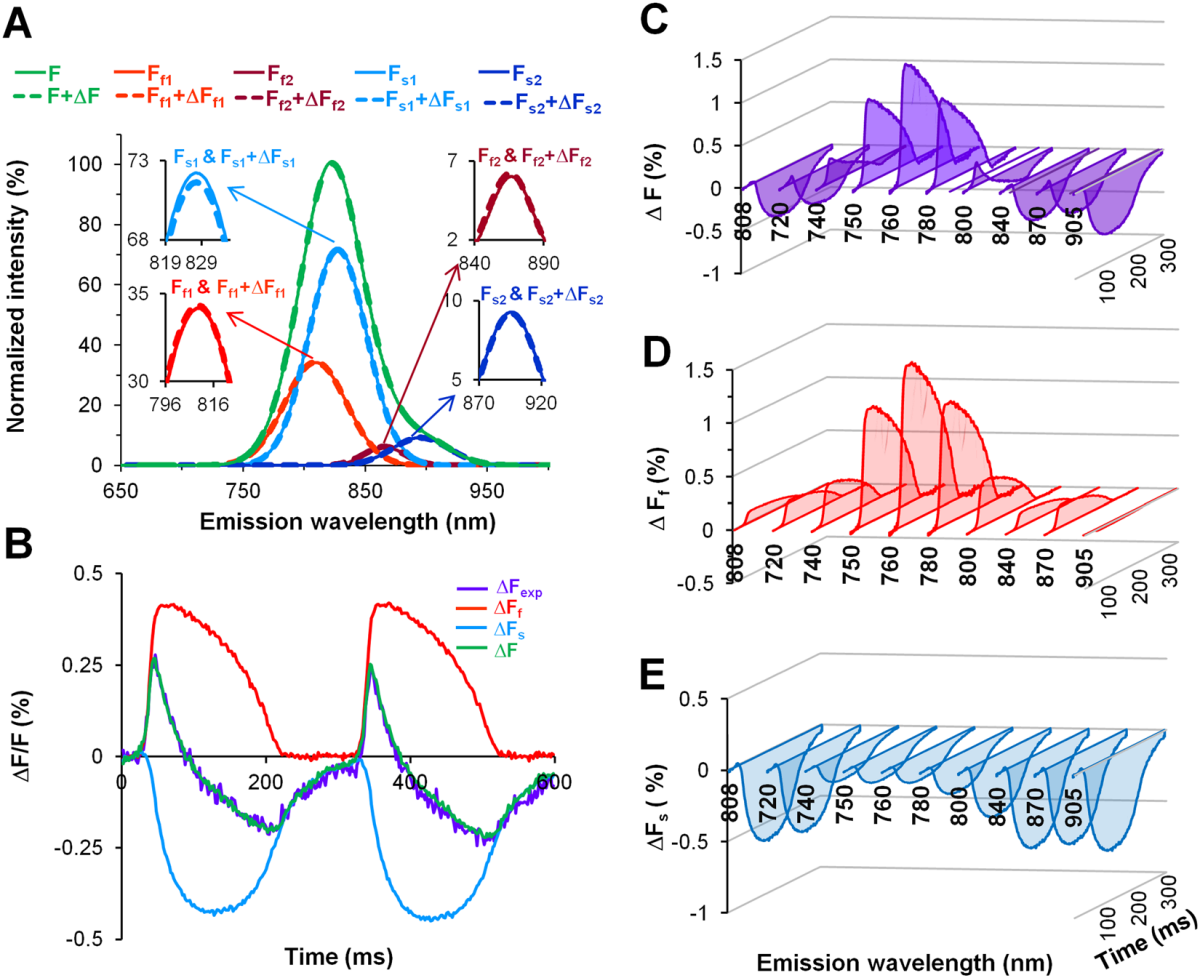
Detection of the emission spectra of the fast and slow components of ICG dye (1 µM) in the heart. (**A**) Superimposition of spectra recorded at λ_ex_ = 660 nm; total fluorescence was normalized to 100%. Four separate spectra obtained at rest (*solid*) and at the maximum amplitude of the AP (*dash*): two for the fast component (F_f1_ and F_f2_; *red* and *dark red*) and two for the slow component (F_s1_ and F_s2_; *blue* and *dark blue*) are presented alongside the calculated total OS (F and F + ΔF, *green*). The spectrum peaks on an expanded fluorescence scale are shown in the insets. (**B**) Superimposition of fractional fluorescence signals at λ_ex/em_ = 660/800 nm. Experimentally detected with a fast EMCCD camera the total voltage-sensitive OS (ΔF_exp_; *violet*), from the total OS calculated fast (ΔF_f_; *red*) and slow (ΔF_s_; *blue*) components, and recalculated OS (ΔF, the sum of ΔF_f_ and ΔF_s_; *green*). (**C–E**) The ICG fluorescence obtained at λ_ex_ = 660 and λ_em_ from 720 nm to 905 nm for the total fractional fluorescence of OS (ΔF; *violet*) and for both components: ΔF_f_ (*red*) and ΔF_s_ (*blue*). The data shown in all panels were obtained in a single experiment.

As mentioned above, to analyze the fluorescence spectra we used four Gaussian profiles, two for each of the two components; again, this may correspond to the fluorescence spectra of ICG-monomeric and of ICG-aggregated forms, as shown in Fig. 2A (F_f1_, F_s1_ and F_f2_, F_s2_, respectively). Figure 2A shows the overlapping spectra for the total OS (*green*) and for each component (two for the fast component, shown in *red* and *dark red*, and two for the slow component, shown in *blue* and *dark blue*) obtained for the resting potential (i.e., the basic fluorescence level; *solid*) along with changes in the spectra that were observed during the action potential (i.e., at depolarization; *dash*), which reflects the sensitivity of the dye to voltage. The difference between the action potential spectrum (*dash*) and the resting spectrum (*solid*) is the voltage-induced fractional change in fluorescence. The data shown in Fig. 2 were recorded at λ_ex_ = 660 nm and at 1 µM ICG. A similar analysis was performed at all tested λ_ex_ and dye concentrations (data not shown).

The voltage sensitive fractions of fluorescence of OS, recorded with a fast EMCCD camera and with corresponding filters for the emission, were calculated by using the ratiometric and multiplication methods^2^. This analysis revealed a dual-component response of the voltage-sensitive signal of the ICG dye. As shown in Fig. 2B, the sum of both calculated components (fast and slow; *red* and *blue*, respectively), which were elicited from a total OS (*violet*), yields a signal (*green*) that corresponds closely to the experimentally obtained fluorescence data (compare *violet vs. green*). Changes in total OS (*violet*) and in calculated a voltage-sensitive OS_f_ (*red*) and OS_s_ (*blue*) components, obtained at λ_ex_ = 660 nm in a full diapason of the ICG fluorescence spectrum, from 740 nm to 905 nm, using band-pass filters for various wavelengths and the long-pass filters (720 nm and 808 nm), are shown in Fig. 2C–E. Notably, all of the OSs spectra obtained in a wide range of fluorescence at rest and their changes during depolarization (Fig. 2A) were calculated and evaluated by estimating both signals recorded simultaneously with a spectrometer and a fast EMCCD camera equipped with the appropriate emission filters.

### Spectral properties of the ICG OS components

**Figure 3:**
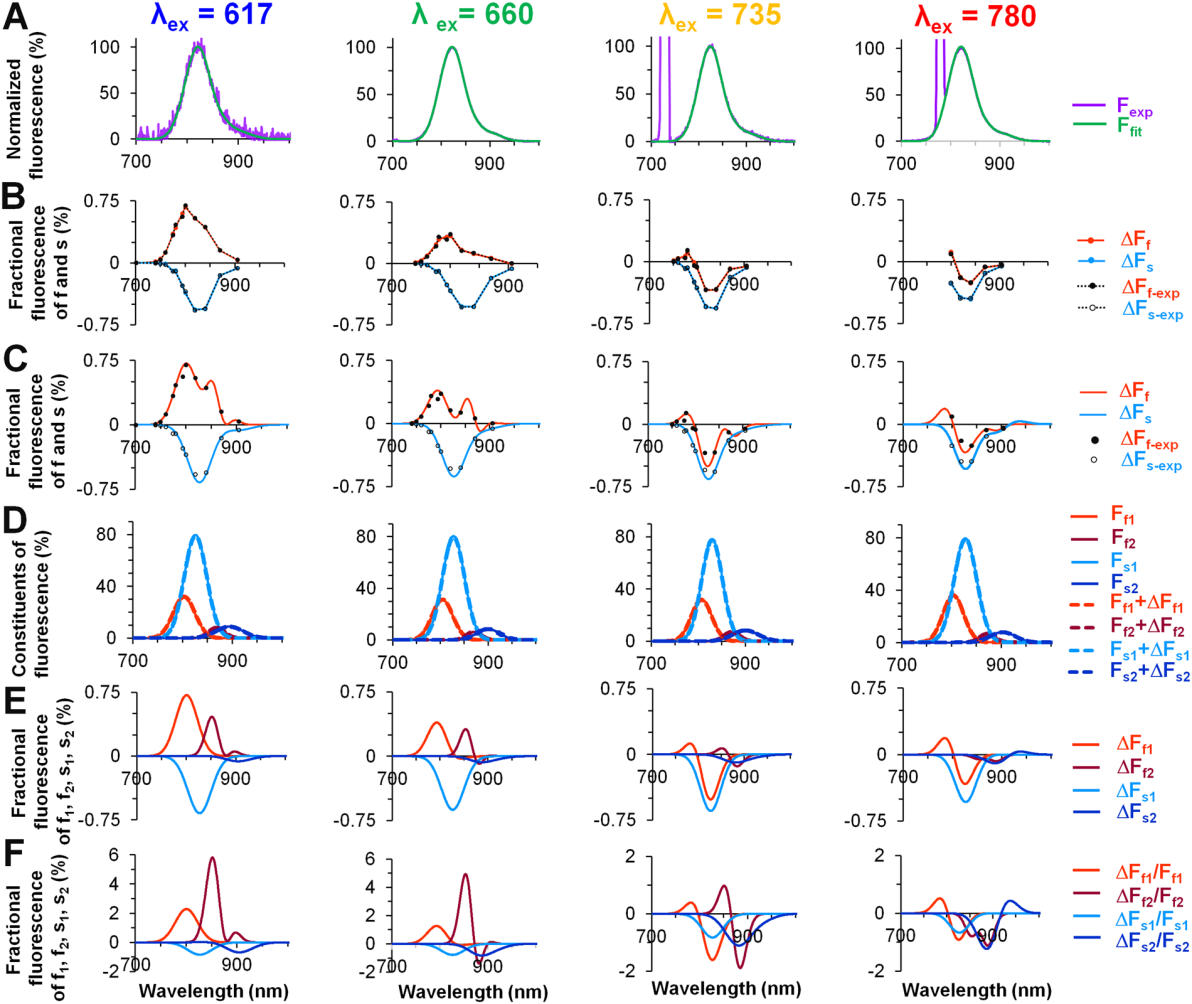
Mean values of the ICG OS fluorescence spectra obtained experimentally or calculated by applying a model with four Gaussian functions. The records shown were obtained at 1 µM ICG (n = 9) and at various excitation wavelengths: λ_ex_ = 617 nm (*blue*), λ_ex_ = 660 nm (*green*), λ_ex_ = 735 nm (*yellow*), and λ_ex_ = 780 nm (*red*). (**A**) The superimposition of normalized emission spectra obtained with a spectrometer (*noisy*) and calculated obtained spectra using a four-Gaussian approximation (*smooth*). (**B**) Two voltage-sensitive components with distinct spectral properties were isolated from the ICG fluorescence spectrum recorded with a fast EMCCD camera at the corresponding emission filters (black circles: *filled* for fast and *open* for slow component) and the calculated values (*red* for fast and *blue* for slow) are shown. (**C**) The same graph as in (**B**) with the calculated data presented as a continuous curve. (**D**) Splitting of the results into four distinct constituents from the spectra presented in (**A**), two by two: fast (*red* and *dark red*) and slow (*blue* and *dark blue*). (**E**,**F**) Fractional changes in all four constituents when the total fluorescence signal, F, is normalized to 100% (**E**) and when the fluorescence of each individual constituent (F_f1_, F_f2_, F_s1_, and F_s2_) is normalized to 100% (**F**).

Next, by comparing the fluorescence recorded with a spectrometer and a fast EMCCD camera and having F, ΔF_f_ and ΔF_s_, we determined which part of the voltage-sensitive fluorescence is proportionally attributable to each of the detected constituents (Fig. 3). Here, we present the analyzed mean data of the ICG (1 µM; n = 9) fluorescence upon excitation using four different LEDs (λ_ex_) 617 nm, 660 nm, 735 nm, and 780 nm. Fitting of the experimental data to a four-symmetrical three-parameter Gaussian function was performed to calculate the total background fluorescence (Fig. 3A, *green*). Under these conditions, this approximation fits the experimental data well (Fig. 3A, *violet*) over the entire range of the ICG dye fluorescence spectrum. In Fig. 3B,C, the indicated points (*black filled* and *unfilled* circles) correspond to the point at which, using the appropriate filters for the emission, the OSs were recorded with a fast EMCCD camera (this procedure is described in more detail in the Methods section). For the spectrometric data, depending on the width and the position of the spectral region passed by the emission filter, the mean value of the spectral region was calculated, compared with the OS value obtained with a fast EMCCD camera (F; background fluorescence) and split into two components (fast and slow; F_f_ and F_s_, respectively, shown in *red* and *blue* in Fig. 3B). The ΔF value has been proportionally adjusted for OS, which has already been differentiated into a fast and a slow component. The fitting procedure with a four Gaussian lineshapes using a least square regression was applied for three experimentally recorded data sets: total fluorescence spectrum recorded with a spectrometer and two fractional fluorescence of fast and slow components. All regression lines well approximate the experimental data with a high correlation coefficient. In average R^2^ for the total fluorescence spectrum and for both voltage-sensitive fractional fluorescence fast and slow components approximations at λ_ex_ = 660 nm and 1 µM of the ICG were 0.999 ± 0.0001, 0.972 ± 0.004, and 0.997 ± 0.001, respectively (for each: n = 9). It is obvious that very good correlations between experimental data and fittings were obtained. Note that Fig. 3C differs from Fig. 3B; in Fig. 3C, the calculated data for ΔF_f_ and ΔF_s_ are presented as continuous curves.

Figure 3D shows the fluorescence spectral lineshapes of the four-symmetrical, three-dimensional Gaussian curves obtained at rest (F) and during depolarization (F + ΔF) for four constituents (F_f1_, F_f2_, F_s1_, F_s2_ and F_f1_ + ΔF_f1_, F_f2_ + ΔF_f2_, F_s1_ + ΔF_s1_, F_s2_ + ΔF_s2_, respectively) with the ICG dye. The fractional fluorescence data (ΔF_f1_, ΔF_f2_, ΔF_s1_, ΔF_s2_) of all four constituents are shown in Fig. 3E. The data presented in Fig. 3A–E, are expressed in percentages based on normalization of the total fluorescence (Fig. 3A) to 100%. Although data presented in Fig. 3F have the same fractional fluorescence for all four constituents as in Fig. 3E, every fluorescence constituent (from Fig. 3D) was normalized to 100%. Therefore, the data in Fig. 3E show the impact of each constituent on the total voltage sensitivity of the dye (f1 and s1 have the largest impact), and the data in Fig. 3F demonstrate the magnitude of the voltage sensitivity of each constituent (f2 is most voltage-sensitive). Besides, in distinct spectral ranges, these constituents have different impacts on the dye’s voltage sensitivity.

### Evaluation of the parameters of Gaussian lineshapes of the ICG OS constituents

**Figure 4:**
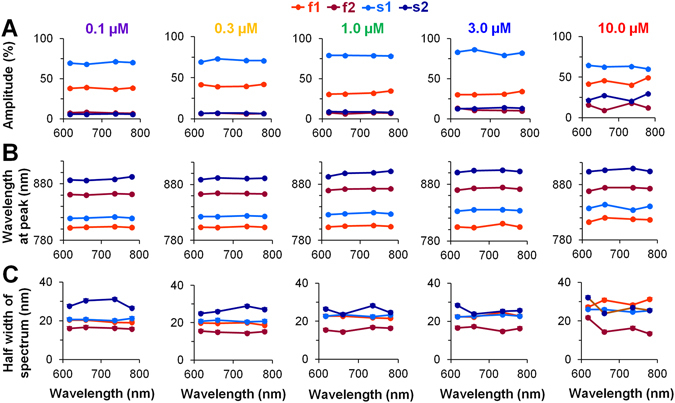
The mean values of Gaussian lineshape parameters for each of the four constituents at various concentrations of the ICG dye. (**A**–**C**) The indicated points in all graphs correspond to excitation at wavelengths of 617 nm, 660 nm, 735 nm, and 780 nm. Each of the four Gaussian constituents (two for fast (f1 and f2; shown in *red* and *dark red*, respectively) and two for slow (s1 and s2; shown in *blue* and *dark blue*, respectively)) is associated with three parameters: the amplitude (**A**), the wavelength at fluorescence maximum (**B**), and the half width of the spectrum at 50% of the fluorescence maximum (**C**). These parameters were measured using five different concentrations of the ICG dye ranging from 0.1 to 10.0 µM (n = 3–9 for each concentration). Error bars indicate s.e.m.

The calculations described above were performed for distinct LEDs and for all tested concentrations of the ICG dye to determine very precisely the parameters for the four Gaussian constituents. The mean values of these parameters are presented in Fig. 4. The amplitude of each constituent at different concentrations of the ICG dye and over a wide range of excitation light wavelengths is shown in Fig. 4A. The amplitude for each constituent was determined by normalizing the total fluorescence intensity to 100%. Under all experimental conditions used in this study, the largest amplitude was obtained for the s1 constituent, which constitutes ~75% of the total fluorescence. The amplitude of the f1 constituent was always smaller than that of s1; it comprised ~30-40% of the total fluorescence amplitude. At λ_ex_ = 660 nm with 1 µM ICG (n = 9) f1 was 31.0 ± 1.4% and s2 was 79.0 ± 2.1%. In general, the amplitudes of the f2 and s2 constituents, which may be related to the presence of ICG-aggregated forms, were always markedly smaller (i.e., only ~10% of the total fluorescence); however there was a tendency for these constituents to increase at higher concentrations (3–10 µM) of the dye.

The concentration dependence of the amplitude of each constituent of OS at four excitation wavelengths is presented in Supplementary Fig. S2. The decrease in the fluorescence intensity of all four constituents at higher ICG dye concentrations could be induced by two different processes: 1) aggregation of the dye molecules followed by self-quenching^17^; and 2) the inner-filter effect^15^, as self-shielding which occurs when the dye itself attenuates the penetration of the excitation light to the deeper myocardial layers and attenuates the emission of the fluorescence from these layers; overall the self-shielding effect weakens the fluorescence signal, and as self-absorption when the reabsorption of emitted light further reduces the fluorescence intensity. However, it is difficult to separate these concentration-dependent processes, especially in biological experiments.

Figure 4B shows the wavelength at the peak of the spectrum for each constituent. It is seen that at different ICG dye concentrations and excitation wavelengths, the obtained difference in λ_max_ between the f1 and s1 constituents at all excitation wavelengths is ~25 nm. At 1 µM ICG and λ_ex_ = 660 nm, the difference in λ_max_ was 22.75 ± 1.42 nm (n = 9); the wavelength for the f1 peak was 804.37 ± 0.95 nm, and the wavelength for the s1 peak was 827 ± 0.57 nm (n = 9 for each); these values were significantly different (*p* < 0.05). Under the same circumstances, the differences in λ_max_ between f1 and f2, along with the differences between s1 and s2, were quite large, i.e., ~60 nm (for f1, f2 the difference was 66.55 ± 1.63 nm, and for s1, s2 it was 72.22 ± 0.64 nm; n = 9 for each). The λ_max_ values for all four constituents at all tested excitation wavelengths were nearly stable at every ICG dye concentration (Fig. 4B), as they were for the total fluorescence spectrum (see Supplementary Fig. S3). The dependence of λ_max_ on ICG dye concentration for f1 and s1 (see Supplementary Fig. S4A), as well as for f2 and s2 (see Supplementary Fig. S4B), is shown as the average value of λ_max_ at all excitation wavelengths. The difference in λ_max_ between f1 and s1 may indicate that the ICG dye is present in diverse environments that have different solvent polarities: for f1, this solvent is of higher polarity, whereas for s1, it is of lower polarity^8^. The large difference in λ_max_ between f1 and f2 also between s1 and s2 likely reflects the formation of separate pools of ICG-monomeric and ICG-aggregated forms. The data allow us to interpret the f1 and s1 constituents as depending on the presence of ICG monomers and f2 and s2 as depending on the presence of ICG-oligomers.

Figure 4C shows the half width of the spectrum at 50% of the peak of the ICG fluorescence constituents. For the ICG-monomeric constituents, f1 and s1, this width is almost the same (~20 nm; compare *red* with *blue*), and at λ_ex_ = 660 nm with 1 µM ICG (n = 9) was 22.7 ± 0.5 nm and 23.5 ± 0.2 nm, respectively. If one considers that f1 and s1 constituents may correspond to the ICG-monomeric form in different environments, the data indicate that the width of the spectrum does not change because of the polarity of the solvent. Interestingly, the width of the spectra of the constituents that can be attributed to the ICG-aggregated form differ from those of the monomeric portion. The observed widening of the s2 constituent (Fig. 4C, *dark blue*) might be explained by different degrees of aggregation of the ICG dye; that is, it could be attributable to the presence of a variety of aggregates (from dimers to polymers). Under these conditions, the shift in the spectrum should also be distinct, and the sum of all of the spectra of molecules with different degrees of aggregation should be widened. However, the width of the spectrum for the f2 constituent, which possibly corresponds to the ICG-aggregated form of the fast component, is narrowed. In general, a narrow spectrum is formed when the fluorescent dye aggregates into J-type aggregates^18^. Nevertheless, we cannot provide an additional argument to support the idea that f2 ICG aggregates correspond to J-type aggregates.

In summary, we suggest that ICG exists in different pools with different spectral characteristics. This point is further explicated in the Discussion section.

**Figure 5:**
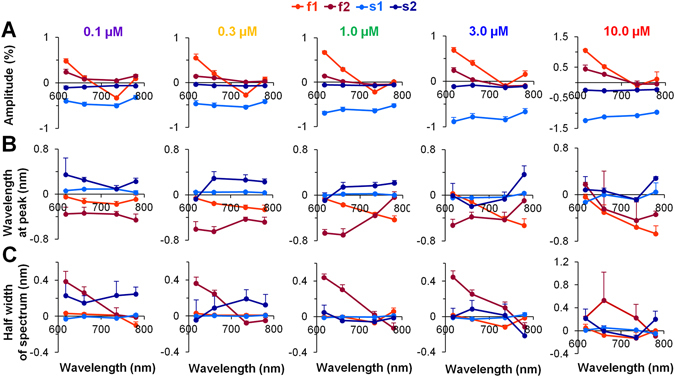
Mean changes in the Gaussian parameters induced by depolarization for each of the four constituents at various concentrations of the ICG dye. The parameters of the fluorescence signals recorded during excitation at wavelengths of 617 nm, 660 nm, 735 nm, and 780 nm are presented as points. (**A**–**C**) Each of the four Gaussian constituents (two for the OS_f_ component (f1 and f2; *red* and *dark red*, respectively) and two for the OS_s_ component (s1 and s2; *blue* and *dark blue*, respectively) has three parameters: the amplitude, the wavelength at fluorescence maximum (i.e., at the peak of the spectrum), and the half width of the spectrum at 50% of the fluorescence maximum. Note: these parameters were determined at five different concentrations of the ICG dye ranging from 0.1 to 10.0 µM (n = 3–9 for each). Error bars indicate s.e.m.

Figure 5 shows the differences from all of the parameters presented in Fig. 4 that appeared as a result of voltage-sensitive changes, i.e., changes induced by the electrical field of the AP. Figure 5A shows the percentage changes in Gaussian lineshape amplitude (ΔF) for each constituent, where 100% represents the maximal value of the total fluorescence. The amplitude of both constituents of the OS_s_ component (s1 and s2, shown in *blue* and *dark blue*, respectively) decreases during depolarization at all excitation wavelengths and at all concentrations of the ICG dye, whereas the changes in the amplitudes of constituents of the OS_f_ component (f1 and f2, shown in *red* and *dark red*, respectively) show a trend that depends on excitation wavelength and can be positive or negative. Usually such changes in the fractional fluorescence amplitude (but symmetrical according to an isosbestic point) appear due to a voltage induced shift in the absorption spectrum when using electrochromic substances^4^.

The various changes in the amplitude of the emission spectrum at particular excitation wavelengths can be explained by the changes in the absorption spectrum. The change in the emission spectrum amplitude for f1 at excitation wavelengths from 617 nm to 780 nm (a gradual change from an increase to a decrease and to an increase again in the amplitude is observed) could be induced by the blue shift of the excitation spectrum, which is specific to electrochromic VSDs. However, the blue-shift of the excitation spectrum should induce symmetrical change in the emission spectrum amplitude according to the isosbestic point (located between 660 nm and 735 nm), and the derivation from the symmetry at 617 nm and 780 nm most likely can be explained by an additional widening of the excitation spectrum. If the blue shift of the absorption spectrum can be explained by the electrochromic feature of the ICG, the widening of the spectrum may reflect the field-induced reorientation of the dye molecules.

The data presented in Fig. 5B show the shift in λ_max_ of the fluorescence spectrum induced by changes in the electrical field. The shift of the spectral peak wavelength of the s1 constituent was close to 0 nm (i.e., 0.013 ± 0.006 nm based on the averaged data obtained at all excitations and all concentrations used) in all of the experimental conditions used in our study. This suggests that there is no shift in the spectrum during depolarization. In contrast, the wavelength peak values obtained for the s2 constituent (the average under all experimental conditions was 0.1 ± 0.01 nm) are of various diverse forms, possibly as the result of difficulties in evaluating the shift in the fluorescence spectrum when the amplitude is so small (see Fig. 4A). It is seen that for the f1 and f2 constituents under all experimental conditions, there was only a negative shift (or a blue shift) in the fluorescence spectrum; this is typical of electrochromic substances.

As shown in Fig. 5C, the width of the ICG-monomeric constituents (f1 and s1) hardly changed during the depolarization. The change in the ICG-aggregated constituents (f2 and s2) is also negligible but because of its large deviation, this value is not reliable. Again, it is very complicated to evaluate such small changes in fluorescence amplitude at the half width of the spectrum.

### Fluorescence of ICG in the presence of proteins and lipids

The maximal peak of the emission spectrum of the fluorescence depends on many factors, including the nature of the solvent, the polarity of the dye molecule, the concentration of the dye, the temperature, the pH, etc. It is known that the absorption/emission properties of the ICG dye in aqueous solution differ from those of the ICG dye in other solvents^16, 19^. Therefore, as an external control, we also investigated the properties of the fluorescence spectrum of the ICG dye at 1 µM in Tyrode’s solution containing BSA or lipids. The fluorescence intensity of ICG increases when the dye molecules bind to proteins and lipids.

We determined that λ_max_ at λ_ex_ = 660 nm in the presence of albumin or lipids was 809.03 ± 2.25 nm and 833.28 ± 3.57 nm, respectively, and that λ_max_ at λ_ex_ = 780 nm was 808.72 ± 2.22 nm and 831.72 ± 2.49 nm, respectively (*p* < 0.05, n = 3 for each). Interestingly, the measured values of λ_max_ in the presence of albumins and lipids are close to the values obtained for f1 and s1 constituents with the ICG dye at 1 µM in the whole rabbit heart (the f1 and s1 constituents at λ_ex_ = 660 nm were 804.37 ± 0.95 nm and 827.56 ± 0.57 nm, respectively, and at λ_ex_ = 780 nm they were 803.79 ± 0.53 nm and 828.0 ± 0.77 nm, respectively; *p* < 0.05, n = 9 for each). Moreover, the difference in λ_max_ in the presence of albumin and lipids is also approximately 25 nm, as we observed in the rabbit heart for the f1 and s1 constituents. Thus, these data arguably support our presumption that the fast component may result from the interaction of the ICG dye with membrane proteins and the slow component may result from the interaction of the dye with the membrane lipid phase.

## Discussion

Expanding on our previous work^2^, in this paper we focused on a detailed exploration of the fluorescence spectrum of ICG dye in the entire rabbit heart and a demonstration of how changes in that spectrum may occur as a result of the existence of voltage-sensitive portions of OS_f_ and OS_s_. Here, we demonstrated that components with distinct time- and voltage-dependent properties also have different spectra. Moreover, we showed that each of the components (fast and slow) is also dual and is composed of two parts: one part of each was attributed to the presence of an ICG-monomeric form and the other to the presence of ICG-aggregated forms. These four separate constituents were distinguished not by accident but because of the unique shapes of their fluorescence spectra, which could be approximated using a summation of symmetrical Gaussian functions. Accordingly, the analytical model that was applied here was based on the isolation of four different Gaussian lineshapes; this allowed us to demonstrate the possible mechanisms that may be responsible for the formation of a voltage-sensitive component of both ICG-monomeric and ICG-aggregated constituents within each component of the ICG OS.

A very complicated situation of ICG dye spectrum formation might occur if the spectrum consists of four parts, each of which has distinct voltage-sensitive properties. The ICG molecule itself is complex, and when it interacts with a multiplex milieu, such as the cell membrane, an intricate fluorescence effect is observed. The ICG dye is a symmetrical, amphiphilic molecule in which two lipophilic polycyclic parts are connected by a long polymethyl chain, a feature that allows the ICG molecule to easily undergo changes in 3D structure^8^. The molecule’s two sulfate residues, which carry a negative charge, are hydrophilic. Depending on the solvent’s properties and heterogeneity, different parts of a molecule of this type could interact with the solvent or with structures present in that solvent, thereby altering its own fluorescence.

Apparently, the IGG molecule does not lose its symmetry in a homogeneous and nonpolar environment and does not form a dipole under such circumstances. It is conceivable that ICG molecules immersed in the nominally homogeneous and nonpolar lipid phase of the membrane behave in this manner. Therefore, we suggest that the chromophore of ICG is aligned with the lipid bilayer surface perpendicular to the membrane electrical field and therefore cannot react electrochromically. Although the ICG molecules in such a lipid pool have no weighty dipole moment, they react to changes in the electrical field because the sulfate moieties at the ends of the molecules are negatively charged. We suggest that the number of ICG molecules in the lipid pool is large enough to result in interaction between the dye molecules, even at low dye concentrations. Such intermolecular interaction is apparent from the concentration-dependent shift of λ_max_ (in Supplementary Figs S1 and S4A). Based on these data, we propose that during cell depolarization the negatively charged molecules in the lipid pool move deeper into the bilayer of the membrane and are squeezed together. Increased intermolecular interaction of the squeezed dye molecules causes self-quenching and decreases the fluorescence intensity of the slow component. The depolarization-induced negative change in the amplitude of the slow component of both constituents (s1 and s2) at all excitation wavelengths is shown in Fig. 5A; there is no appreciable change either in the position of the spectrum peak (Fig. 5B) or in its width (Fig. 5C). Therefore, we propose that the voltage sensitivity of the slow component is produced by a field-induced squeezing of ICG molecules within the lipid environment of the cell membrane that increases the intermolecular interaction, leads to self-quenching, and decreases the fluorescence intensity of both the s1 and the s2 constituents. Arguably, the slow component (both the s1 and the s2 constituents) is produced by ICG molecules that are surrounded by membrane lipids.

Biological membranes contain polar compounds, such as proteins. The hydrophilic parts of ICG molecules can interact with the charged radicals of membrane proteins, whereas the lipophilic parts of the dye molecule may be immersed in the lipid phase of the membrane. Thus, the dye molecules anchored to the proteins in the membrane might lose their symmetry with respect to the surface of the membrane, and a dipole that reacts to changes in the electric field within the membrane could be formed. In this way, the ICG molecule would acquire voltage sensitivity. That voltage sensitivity could be of two types: (I) an electrochromic type in which the charge transition in the chromophore reacts to changes in the electric field; and (II) a reorientation in which, as the result of a change in the membrane potential, the depth to which a freely floating end of the dye molecule is immersed in the lipid phase of the membrane changes and its dipole moment is thereby changed. The analysis of voltage-induced changes in the fast constituent of the ICG-monomer (f1) shows that depolarization shifts the fluorescence spectrum to the left (Fig. 5B) without changing its width (Fig. 5C). Although we did not record the full excitation spectrum in this study, we obtained fluorescence spectra at four different excitation wavelengths. Therefore, our analysis of voltage-sensitive changes in the amplitude of fluorescence spectra recorded at these four excitations (see Fig. 5A) allowed us to suggest that among other changes, depolarization induces a left shift of the excitation spectrum. Additionally, we suggest that widening of the spectrum occurs. Taken together, our data allow us to propose that the voltage sensitivity of the f1 constituent is formed by the electrochromic mechanism together with the reorientation of the ICG molecules during generation of the AP.

At increased dye concentrations, ICG molecules that are anchored to proteins and thus have weighty dipole moments can interact with each other to form aggregates. In this study, we describe these aggregates as f2. We further suggest that these aggregates may be of J-type^18^. This idea is supported by the observation that the spectrum width of the f2 constituent is reduced (see Fig. 4C). This type of aggregate, which lacks symmetrical charge distribution in a chromophore, has its own characteristic electrochromic features, including a depolarization-induced a left shift of the excitation (see in Fig. 5A the gradual excitation-wavelength-dependent decrease in the amplitude of the f2 constituent) and emission (Fig. 5B) spectra.

Depolarization-induced broadening of the fluorescence spectrum but not of the excitation spectrum was found for di-4-ANEPPS, in addition to an electrochromic blue shift of both spectra. The phenomenon named as “field induced resolvation” in the excited state of the dye was proposed to be responsible for the broadening of the fluorescence spectrum^9^. In contrast, we suggest that depolarization induces broadening of the excitation spectrum but not the fluorescence spectrum of ICG, in addition to an electrochromic shift of both spectra; we further propose that in the ground state field-induced movement of the lipophilic fraction of the ICG molecules anchored to proteins is responsible for that broadening. Arguably, these additional features of both VSDs can be attributed to the field-induced reorientation of the dye molecules within the membrane^20^.

Finally, we propose that the voltage sensitivities of the fast and slow components of the ICG-induced fluorescence signal occur by completely different mechanisms.

The ICG-aggregated fast and slow constituents, according to our experimental data, are also voltage-sensitive, and their mechanisms may not differ markedly from those that occur in the ICG-monomeric constituents.

## Summary and future directions

This study demonstrates that because of its very complex molecular structure, ICG may exist in different pools in heart cell membranes, each of which has distinct spectral characteristics and acts via a separate voltage-sensitive mechanism. We considered numerous possible mechanisms for the voltage sensitivity of the dye, including electrochromism, field-induced reorientation, and field-induced squeezing of dye molecules, all of which might simultaneously play a role in depolarization-induced changes in dye fluorescence in the heart. The characteristics of ICG fluorescence that account for its voltage sensitivity in cardiac imaging may be further optimized.

Certainly more clarity would be obtained through further exploration of both the excitation and emission spectra of the dye, and this will be our future direction. We believe that this report represents an important step that may help provide a future basis for introducing the optical mapping of cardiac electrical activity in the clinic.

## Limitations

One limitation of our study is that faithful and precise detection of the voltage-sensitive mechanisms of the dye could be obtained only when both excitation and emission spectra were recorded. Another limitation may be related to the calculation of OSs; in the analysis, signals were taken not from an area of 5 × 5 pixels, which would permit evaluation of the kinetics of the AP^3^, but from a much larger area (15 × 40 pixels) corresponding to about 10 ms of propagation time. Hence, the kinetics of OSs were not investigated. The averaging of signals obtained over a period of approximately 10 ms had no noticeable impact on the investigated parameters of the fluorescence spectrum during depolarization but reduced the noise associated with the signal.

## Electronic supplementary material

Supplementary Information
