# Supplementary material for: Spectral characteristics of voltage-sensitive indocyanine green fluorescence in the heart
Source: Sci Rep. 2017 Aug 11;7:7983. doi: 10.1038/s41598-017-08168-7 (PMC5554165; doi:10.1038/s41598-017-08168-7)
Supplement: Supplementary file 1 — Supplementary Information [file 41598_2017_8168_MOESM1_ESM.doc]

**Supplementary Information**

**Spectral characteristics of voltage-sensitive indocyanine green fluorescence in the heart**

Regina Mačianskienė,^1^, Mantė Almanaitytė,^1^ Rimantas Treinys,^1^ Antanas Navalinskas,^1^ Rimantas Benetis,^1^ Jonas Jurevičius^1*^

^1^Institute of Cardiology, Lithuanian University of Health Sciences, Kaunas, Lithuania

*Correspondence: [jonas.jurevicius@lsmuni.lt](mailto:jonas.jurevicius@lsmuni.lt)


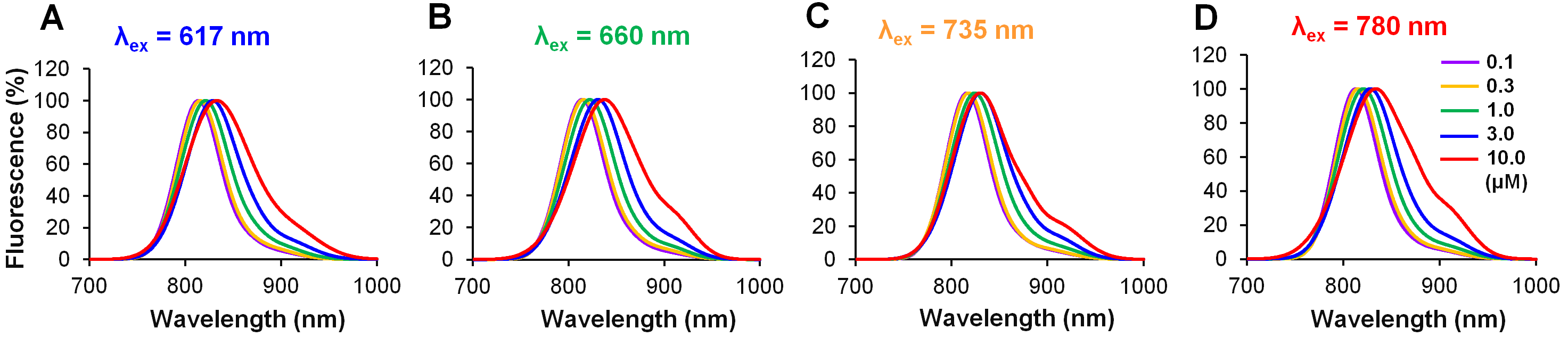


**Figure S1**. **Concentration dependent changes in** **ICG fluorescence spectrum at four different excitation wavelengths used in the study in Langendorff-perfused rabbit heart.** (**A-D**) Average of normalized fluorescence spectra at λ_ex_=617 nm, λ_ex_=660 nm, λ_ex_=735 nm, and λ_ex_=780 nm (n=3-7 for each). (**D**) Insert – ICG dye concentrations as indicated (in µM). Note the shift in the fluorescence spectrum.


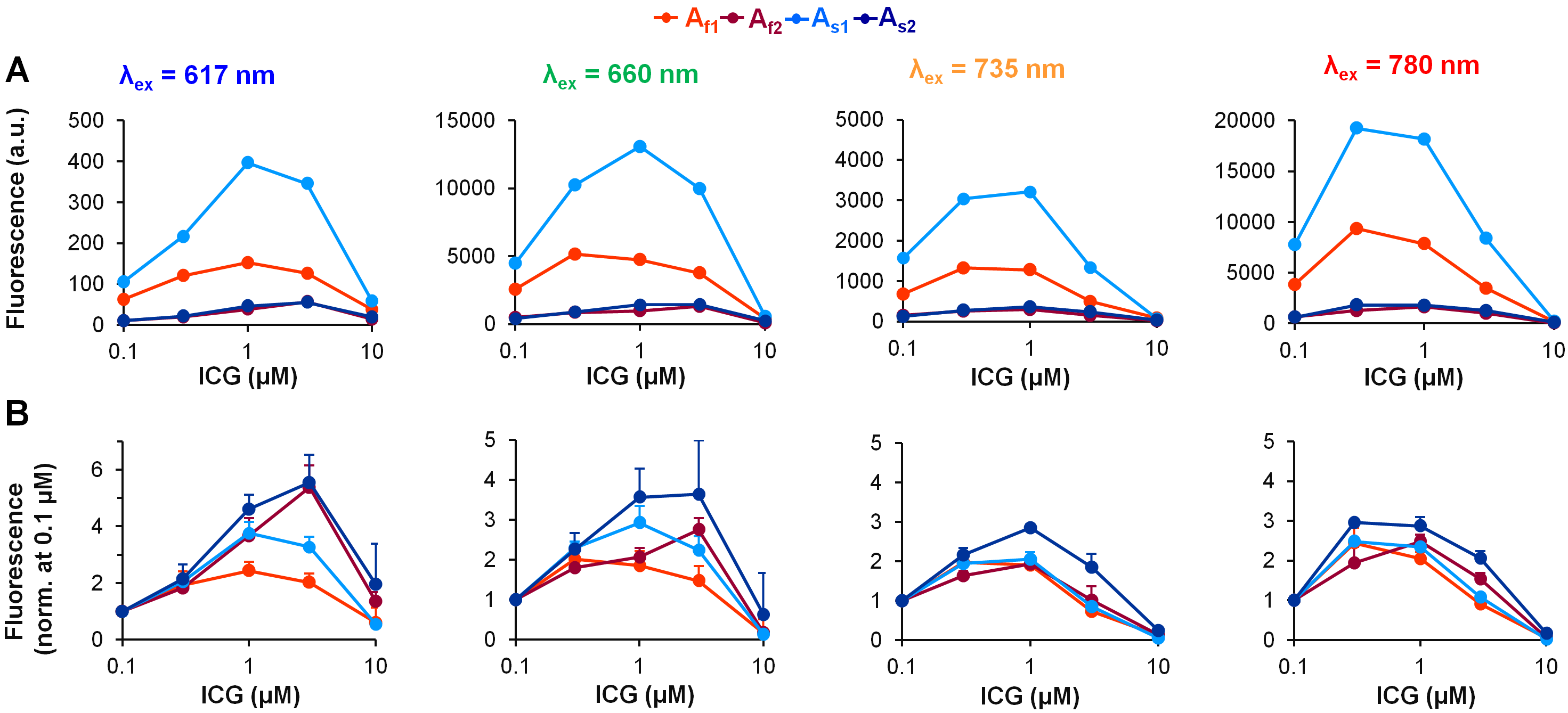


**Figure S2. Variations in the ICG fluorescence spectral amplitude of all four constituents as a function of dye concentration.** (**A-B**) Mean amplitudes (n=3-7) of the emission peaks at increased concentrations of ICG dye: fluorescence intensity in arbitrary units (a.u.) and after fluorescence normalization (at 0.1 µM ICG) at excitation wavelengths of 617 nm, 660 nm, 735 nm, and 780 nm. At the top of the figure, the amplitude of each constituent is indicated (A_f1_, *red*; A_f2_, *dark red*; A_s1_, *blue*; A_s2_, *dark blue*).


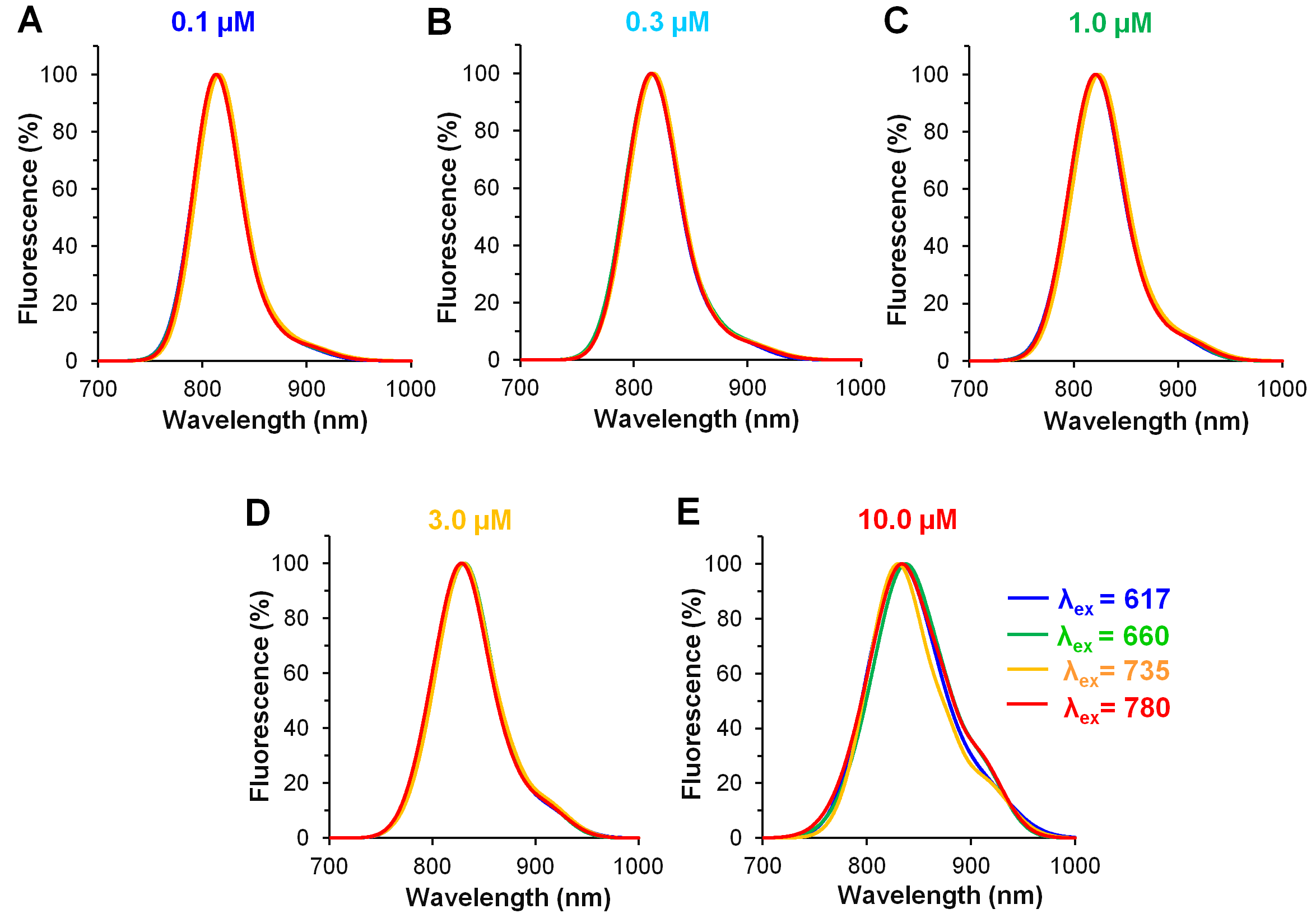


**Figure S3**. **Excitation-dependent changes in ICG fluorescence at five dye concentrations used in the study in Langendorff-perfused rabbit heart.** (**A-E**) Average of normalized fluorescence spectra at increasing ICG dye concentrations (from 0.1 to 10.0 µM) (n=3-7 for each). (**E**) Insert – λ_ex_=617 nm (*blue*), λ_ex_=660 nm (*green*), λ_ex_=735 nm (*yellow*), and λ_ex_=780 nm (*red*). Note: no shift in the fluorescence spectrum occurs at the different excitation wavelengths used.


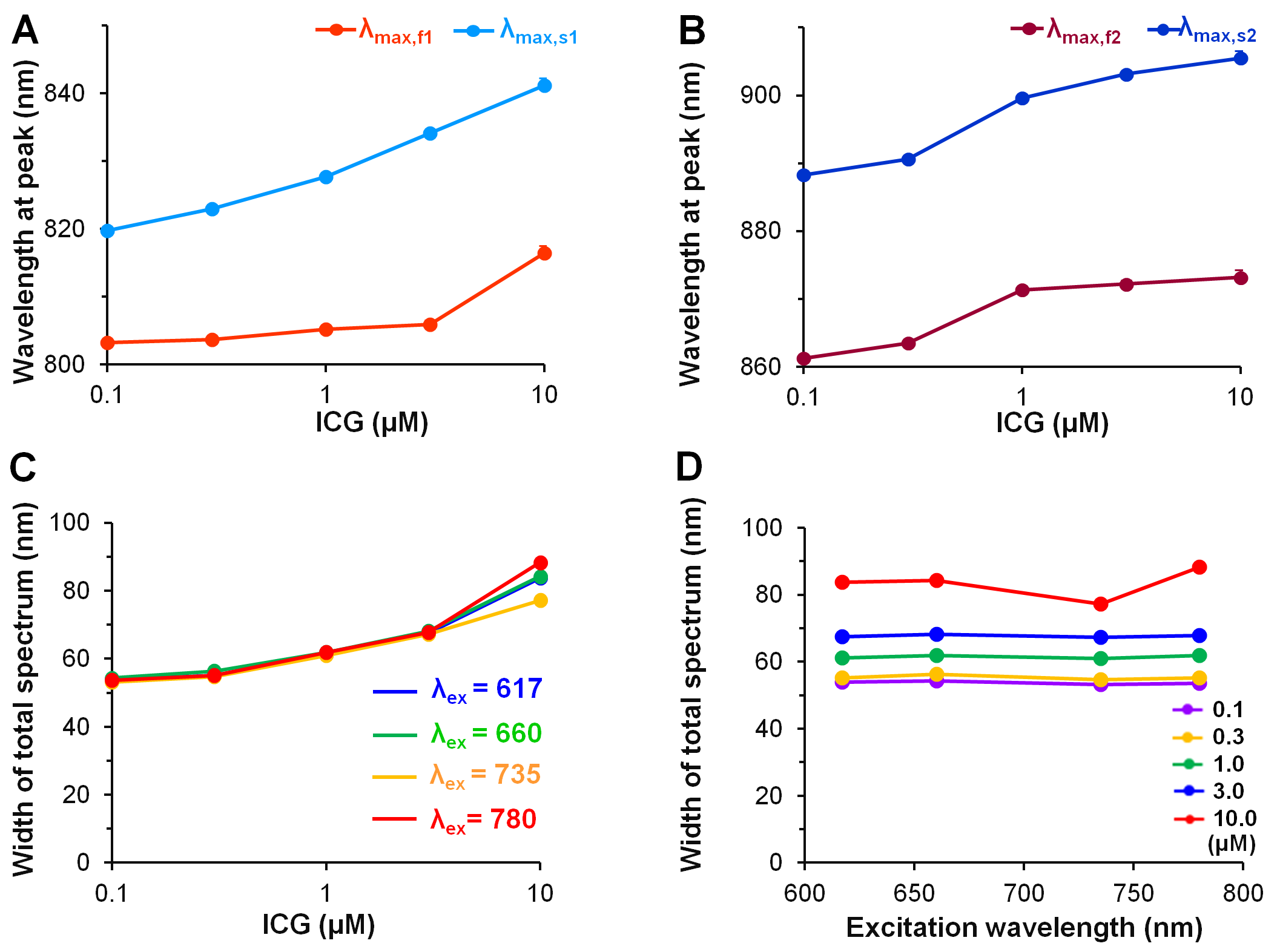


**Figure S4**. **Impact of concentration and excitation wavelength on the ICG fluorescence peak and width of the spectra of the four constituents.** (**A-B**) Dose-dependent effects of the dye on the ICG‑monomeric (f1 and s1) and ICG-aggregated (f2 and s2) constituents, respectively. Note: intermolecular interaction of the fluorescence in the s1 pool occurs at increasing ICG dye concentrations. (**C-D**) Changes in the width of the fluorescence spectrum at four excitation wavelengths and at different ICG dye concentrations, as indicated in the inserts.

| **LED** | **Filter** | **CWL**  **(nm)** | **FWHM**  **(nm)** | **The Company** |
| --- | --- | --- | --- | --- |
| M617L3 | FF01-618/26-25 | 618 | 30.7 | Semrock |
| M660L3 | FBH650-40 | 650 | 40 | ThorLabs |
| M735L3 | FL730-10 | 730 | 10 | ThorLabs |
| M780L3 | FBH780-10 | 780 | 10 | ThorLabs |

## Table S1. Features of the LEDs and excitation bandpass filters. All LEDs from ThorLabs. Abbreviations: CWL - Center Wavelength, FWHM - Full Width Half Max (Bandwidth). Diameter of all filters - 25 mm.

| **Filter** | **Filter type** | **CWL**  **(nm)** | **FWHM (nm)** | **The Company** |
| --- | --- | --- | --- | --- |
| FB700-40 | bandpass | 700 | 40 | ThorLabs |
| FF01-740/13-25 | bandpass | 740 | 19.9 | Semrock |
| FB750-10 | bandpass | 750 | 10 | ThorLabs |
| FF01-760/12-25 | bandpass | 760 | 20.2 | Semrock |
| FF01-775/46-25 | bandpass | 775 | 54.7 | Semrock |
| FBH780-10 | bandpass | 780 | 10 | ThorLabs |
| FF01-794/32-25 | bandpass | 794 | 39.9 | Semrock |
| FF01-800/12-25 | bandpass | 800 | 19.9 | Semrock |
| FF01-819/44-25 | bandpass | 819 | 58.6 | Semrock |
| FF01-840/12-25 | bandpass | 840 | 21.1 | Semrock |
| 870nm CWL , 10nm FWHM | bandpass | 870 | 10 | Edmund Optics |
| FL905-25 | bandpass | 905 | 25 | ThorLabs |
| HQ720LP | longpass | 720* | - | Chroma |
| BLP01-808R-25 | longpass | 823* | - | Semrock |

## Table S2. Features of the emission filters. Abbreviations: CWL - Center Wavelength, FWHM - Full Width Half Max (Bandwidth); * indicates wavelength at 50% of edge. Diameter of all filters - 25 mm.
